# A Short Double-Stapled Peptide Inhibits Respiratory Syncytial Virus Entry and Spreading

**DOI:** 10.1128/AAC.02241-16

**Published:** 2017-03-24

**Authors:** Vanessa Gaillard, Marie Galloux, Dominique Garcin, Jean-François Eléouët, Ronan Le Goffic, Thibaut Larcher, Marie-Anne Rameix-Welti, Abdelhak Boukadiri, Julien Héritier, Jean-Manuel Segura, Elodie Baechler, Miriam Arrell, Geneviève Mottet-Osman, Origène Nyanguile

**Affiliations:** a HES-SO Valais, Sion, Switzerland; b VIM, INRA, Université Paris-Saclay, Jouy-en-Josas, France; c Department of Microbiology and Molecular Medicine, University of Geneva School of Medicine, CMU, Geneva, Switzerland; d INRA, UMR 703 APEX, Oniris, Nantes, France; e UMR INSERM U1173 I2, UFR des Sciences de la Santé Simone Veil—UVSQ, Montigny-Le-Bretonneux, France; f AP-HP, Laboratoire de Microbiologie, Hôpital Ambroise Paré, Boulogne-Billancourt, France; g UMR INRA, Génétique Animale et Biologie Intégrative, Equipe plateforme @BRIDGE, plateau d'histologie, Jouy-en-Josas, France

**Keywords:** respiratory syncytial virus, stapled peptides

## Abstract

Synthetic peptides derived from the heptad repeat (HR) of fusion (F) proteins can be used as dominant negative inhibitors to inhibit the fusion mechanism of class I viral F proteins. Here, we have performed a stapled-peptide scan across the HR2 domain of the respiratory syncytial virus (RSV) F protein with the aim to identify a minimal domain capable of disrupting the formation of the postfusion six-helix bundle required for viral cell entry. Constraining the peptides with a single staple was not sufficient to inhibit RSV infection. However, the insertion of double staples led to the identification of novel short stapled peptides that display nanomolar potency in HEp-2 cells and are exceptionally robust to proteolytic degradation. By replacing each amino acid of the peptides by an alanine, we found that the substitution of residues 506 to 509, located in a patch of polar contacts between HR2 and HR1, severely affected inhibition. Finally, we show that intranasal delivery of the most potent peptide to BALB/c mice significantly decreased RSV infection in upper and lower respiratory tracts. The discovery of this minimal HR2 sequence as a means for inhibition of RSV infection provides the basis for further medicinal chemistry efforts toward developing RSV fusion antivirals.

## INTRODUCTION

Lower respiratory infection is one of the leading causes of human death worldwide (1) and is the most important cause of mortality in infants. Among the pathogens responsible for these infections, human respiratory syncytial virus (RSV) accounts for approximately 20% of all lower respiratory infections in infants (2). The global incidence of infant mortality due to RSV is the highest in developing countries, and although it is much lower in developed countries, it is a high burden on the health care system because of the large number of children that must be hospitalized. RSV can also cause fatal respiratory tract infections in fragile or immunocompromised individuals. Recently, RSV has been recognized as a significant cause of severe respiratory infection in the elderly. In a study performed in the United States (3), the mortality rates were found to be higher in the elderly than in children. No vaccine is presently available against RSV, as it appears to be extremely difficult to immunize patients without inducing immunopathological responses, especially in the very young population. Treatment options are limited to a costly prophylactic treatment of at-risk infants with the monoclonal antibody (MAb) palivizumab (Synagis) and to controversial therapeutic intervention with the nucleoside analog ribavirin (Rebetol) (4). Thus, there is a need for a more effective treatment for the at-risk population.

The RSV fusion glycoprotein (F) is the focus of active research in the field of vaccines, neutralizing antibodies, and small molecules. The antiviral compounds currently investigated in clinical settings are the two small molecules GS-5806 (50% effective concentration [EC_50_] = 0.4 nM) (5) and JNJ-53718678 (6) and ALX-0171, a trimeric nanobody that binds to the antigenic site II of F with subnanomolar affinity (7). RSV F is a type I fusion protein that is activated into a metastable prefusion state upon cleavage by host proteases. The prefusion state contains a hydrophobic fusion peptide, two heptad repeats (HR1 and HR2), and a viral transmembrane domain. Following the insertion of the fusion peptide into the target cell membrane, F undergoes a dramatic conformational rearrangement to yield a six-helix bundle hairpin structure, whereby 3 HR2 sequences bind in an antiparallel manner to a trimeric HR1 coiled coil. The hairpin structure brings the viral and target cell membranes into close apposition, thereby facilitating membrane fusion and subsequent viral entry (8, 9). Peptides derived from these domains can function as dominant negative inhibitors by binding to the transiently exposed coiled coil in the prehairpin fusion intermediate. The resemblance of the folding of these two RSV HR domains with the HIV fusion mechanism of gp41 suggests that it should be possible to develop RSV peptide therapeutics similar to enfurvitide and analogs (10, 11). This strategy was investigated previously by scanning synthetic peptides of 35 amino acids (aa) in length across the RSV HR2 wild-type sequence, resulting in the identification of peptides capable of blocking virus syncytium formation, e.g., T108 and T118 (12). The authors did not report further progress, perhaps because of the poor inhibitory potencies of this class of peptides compared to the HIV analogs. This may be due to a weak affinity of the HR2 peptides for the trimeric HR1 fusion intermediate. Alternatively, it has been suggested that the weak potency may also be due to differences in the fusion kinetics (13). A well-known strategy to bypass this issue consists in stabilizing the α-helical nature of the peptide in the unbound state, by chemical cross-linking of amino acid side chains that are not interacting with the target, thereby decreasing the entropic cost for binding to the target (14). This was successfully achieved by constraining short RSV HR2 peptides with cross-lactam bridges (15). However, these compounds are not druggable, because of the nature of the cross-linking bridges, which are susceptible to proteolytic degradation in the plasma. Recently, the stapled-peptide chemistry technology has emerged as a promising tool to solve this issue (16, 17) and has been used to synthesize constrained peptides derived from T118, an HR2 segment of 35 residues (18). Nonnatural olefinic amino acids are included in the peptides, and the olefinic side chains are cross-linked by ruthenium-catalyzed metathesis. The stapled-peptide chemistry can increase dramatically the potency, proteolytic stability, and cell permeability of the peptide (19).

Here, we have performed a stapled-peptide scan to identify an HR2-derived minimal sequence that interferes efficiently with the formation of the six-helix bundle postfusion structure required for viral cell entry. We describe the discovery of short double-stapled peptides, which display potent antiviral activity and low susceptibility to proteolysis, and demonstrate that intranasal delivery of the peptide identified here to BALB/c mice can efficiently prevent RSV infection.

## RESULTS

### Stapled-peptide scan across the HR2 domain.

**FIG 1:**
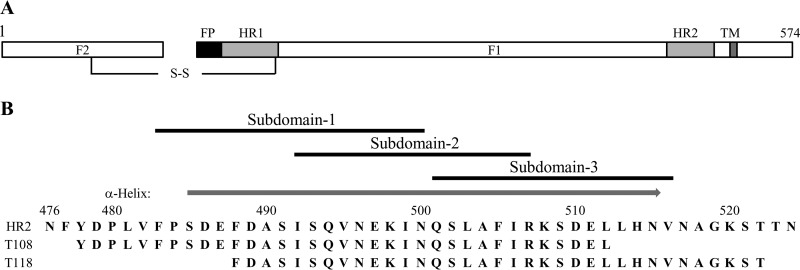
(A) Schematic representation of the fusion protein F. The F1 chain is linked to the F2 chain via a disulfide bridge. FP, fusion peptide; HR1, heptad repeat 1; HR2, heptad repeat 2; TM, transmembrane domain. (B) Stapled-peptide walk. The overlapping subdomains 1, 2, and 3 that were screened are shown above the full-length sequence of the HR2 domain. A gray arrow above the HR2 sequence indicates the region that was observed as an α-helix in the X-ray structure of the postfusion structure (PDB accession number 1G2C). T108 and T118 are the reference peptides used in this study (12).

**FIG 2:**
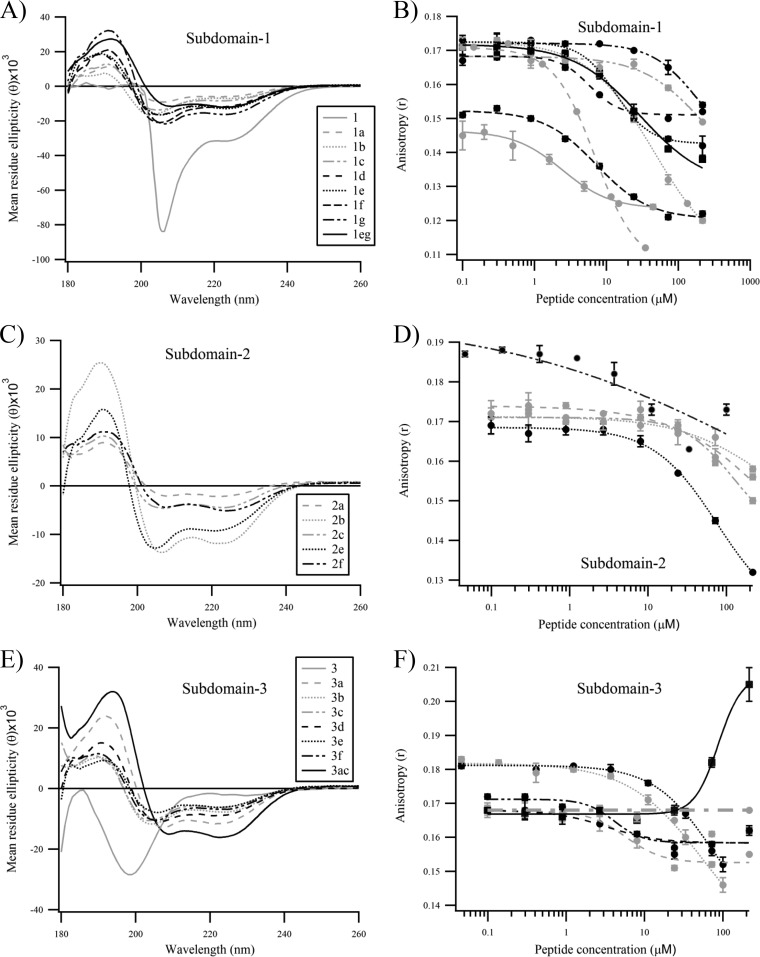
Biochemical characterization of the stapled peptides tested in this study. (A, C, and E) Circular dichroism spectra of stapled peptides derived from subdomains 1, 2, and 3, respectively. (B, D, and F) 5HB fluorescence polarization competition assay performed with stapled peptides derived from subdomains 1, 2, and 3, respectively. The curves were fitted in Igor Pro using the Hill equation function. Error bars are standard deviations from duplicates.

The RSV HR2 region (residues 476 to 524) is a 49-amino-acid sequence that has been extensively characterized (Fig. 1). HR2 is a largely unstructured peptide in aqueous solution folding into an α-helix upon binding to trimeric HR1 coiled coils (20). X-ray structure analysis revealed that only part of HR2 (aa 485 to 515) folds into an α-helix (8). To identify short peptides capable of interfering with 6-helix bundle formation, we designed 16-mer stapled peptides derived from HR2. We reduced HR2 (aa 483 to 516) into three short overlapping subdomains (subdomains 1 to 3) (Fig. 1). These peptides were scanned for various stapling combinations. The residues that are not interacting with trimeric HR-1, as seen in the X-ray structure coordinates, were replaced by nonnatural olefinic amino acids (Table 1). Far-UV circular dichroism (CD) spectropolarimetry was used to investigate the effect of stapling on the α-helical content of the peptides. Similarly to full-length HR2, the native peptides 1 and 3 corresponding to each subdomain (Table 1; Fig. 2A and E) appeared to be largely unfolded, with a characteristic minimum at 200 nm. Peptide 2 could not be investigated in this study, as it was found to be insoluble in all solvents tested. As expected, stapling conferred a significant enhancement of α-helical content to all peptides as observed by the displacement of the random coil minimum toward 208 nm and the appearance of a second minimum at 222 nm (Fig. 2A, C, and E). The α-helical content varied significantly among the peptides and ranged from 5.2% to 47%. Interestingly, the double-stapled peptides 1eg and 3ac both displayed a θ_208_/θ_222_ molar ellipticity ratio close to 1, another indicator of α-helical stability, thereby suggesting that double stapling confers an increased stability to these molecules. To assess the propensity of the stapled peptides to hinder the formation of the postfusion six-helix bundle required for viral cell entry, we used a 5-helix bundle (5HB) biochemical fluorescence polarization (FP) competition assay as previously described (21, 22). Briefly, each stapled peptide was assessed for its ability to compete with the binding of an HR2 fluorescently labeled peptide (T108) to 5HB, a recombinant protein containing 3 HR1 domains covalently attached to 2 HR2 domains. In contrast to the beneficial effect observed in the circular dichroism studies, stapling did not improve the binding affinity of the studied peptides to 5HB. In many instances, stapling appeared to be detrimental to the binding (Fig. 2B, D, and F). Only peptides 1a, 1d, 1f, 3a, 3d, and 3f displayed a binding affinity that was similar to that of the native peptide 1 (Table 1). Unexpectedly, we could not assess the inhibitory activity of the double-stapled peptide 3ac in this assay, because the polarization signal increased instead of decreasing in the presence of the competitor (Fig. 2F). We found that this artifact was due to nonspecific binding of peptide 3ac to the fluorescently labeled probe only, which most likely results in an oligomer inducing a higher polarization than the 5HB/T108 bound complex (data not shown).

**TABLE 1:**
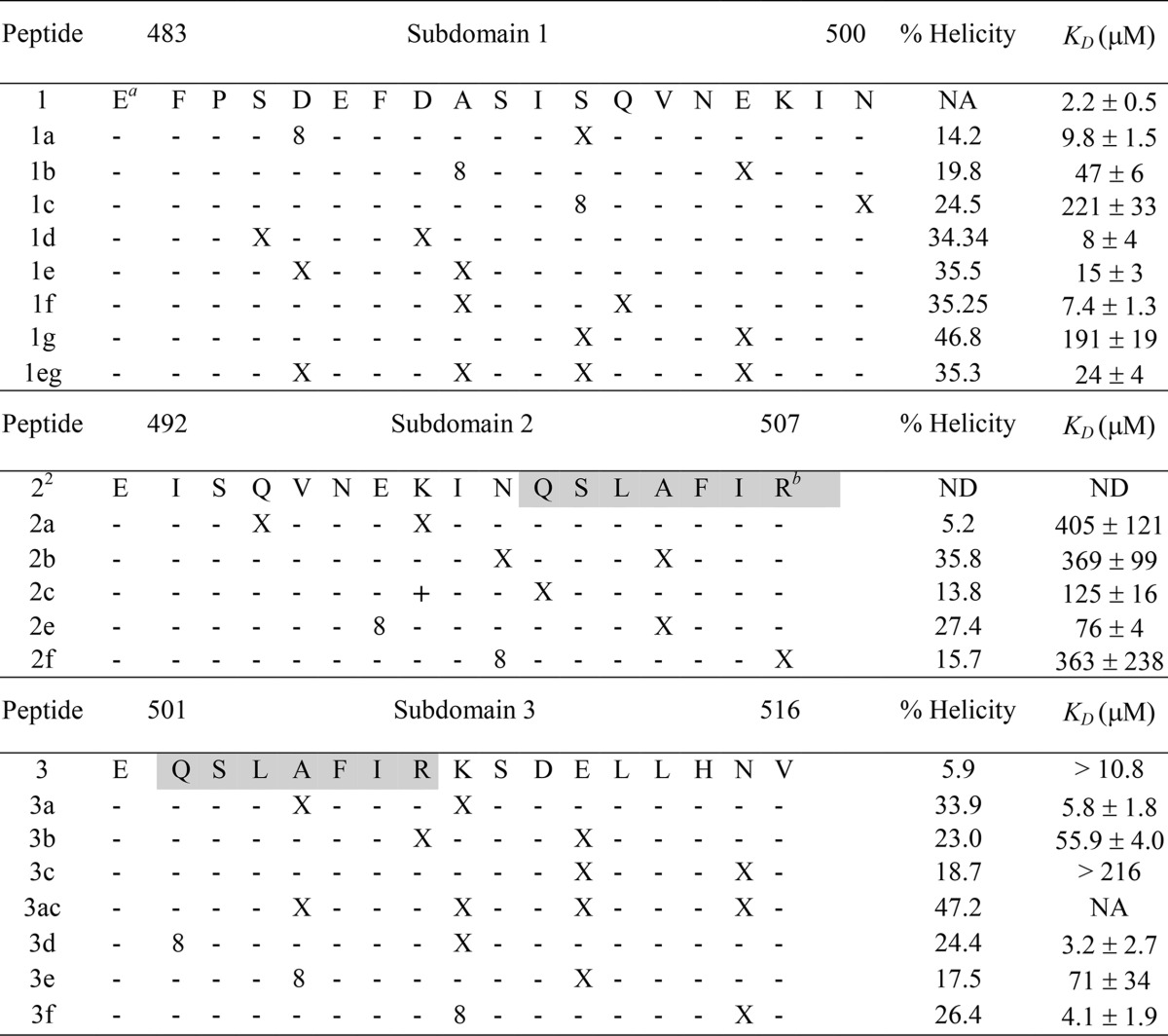
Amino acid sequences, α-helical contents, and *K_D_* values of stapled peptides derived from subdomains 1, 2, and 3^*c*^

^a^The N-terminal Glu residue is not present in the HR2 native sequence; Glu was added to each subdomain to stabilize the macrodipole of the α-helix.

^b^The shaded area highlights the overlapping regions of subdomains 2 and 3.

c 8, R-octenyl-alanine; X, S-pentenylalanine; +, R-pentenylalanine; *K_D_*, equilibrium dissociation constant; NA, not applicable; ND, not determined.

### Identification of short stapled-peptide inhibitors of RSV fusion.

**FIG 3:**
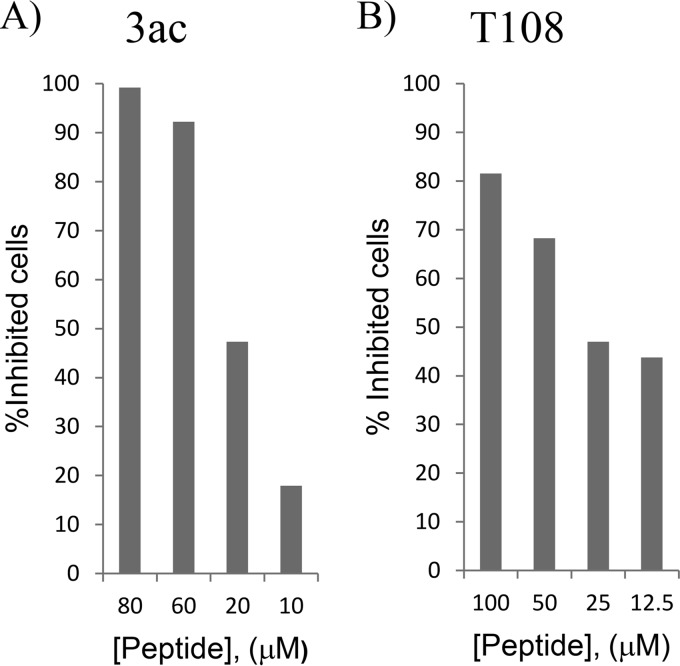
Inhibition of RSV infection by double-stapled peptide 3ac (A) and unstapled peptide T108 (12) (B) in A549 cells. Each peptide was diluted 3 times by 2-fold serial dilutions. eGFP RSV virus at an MOI of 0.5 was used for the infection, and GFP-positive cells were counted by FACS analysis 24 h p.i. The GFP data were normalized to the fluorescence values observed with virus only and plotted as percent inhibition.

Because of the high alpha-helical content that was observed for the double-stapled peptide 3ac in the circular dichroism studies, we decided to assess its inhibitory activity in a cellular viral infectivity assay. A549 cells were infected with a recombinant green fluorescent protein (GFP)-expressing RSV (strain A2) (23), and the inhibitory activity of the peptide was quantified by flow cytometry. We found that peptide 3ac was capable of blocking viral infection of cells similarly to T108 (Fig. 3), whereas none of the best single-stapled peptides, 1a, 1b, 1d, 1f, 3a, 3d, and 3f, identified in the FP biochemical assay, were capable of inhibiting viral infection (data not shown). Altogether, these data suggest that double stapling is required to achieve antiviral potency within this series of stapled peptides. These findings led us to focus on double-stapled peptides derived from peptide 3ac.

### Optimization of the lead stapled peptide.

**FIG 4:**
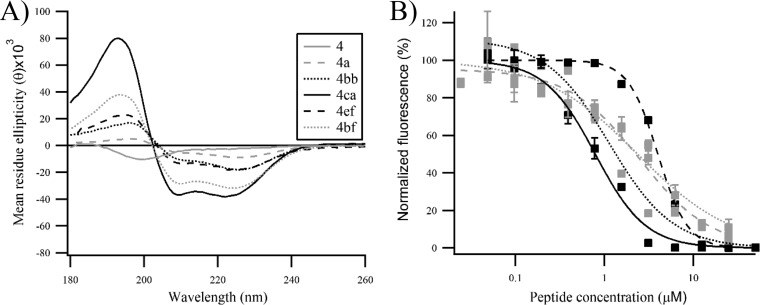
Effects of double-stapling optimization of peptide 4. (A) Circular dichroism analysis of the double-stapled peptides showing a significant conformational change from unstructured peptide 4 to constrained double-stapled peptide 4. (B) Inhibition of RSV infection in HEp-2 cells by optimized double-stapled peptides. Peptides 4ca and 4bb display the most robust viral inhibitory activity. m-Cherry RSV at an MOI of 0.2 was used for the infection, and the mCherry fluorescence was measured 48 h p.i. Error bars are standard deviations from duplicates.

In order to improve peptide potency, we first increased the length of peptide 3ac on the N terminus up to Glu497 to increase aqueous solubility and favor the macrodipole of the α-helix (16), resulting in peptide 4a (Table 2). The CD spectrum of the native peptide 4 showed a pattern consistent with a largely unstructured peptide (Fig. 4A). Interestingly, peptide 4a displayed a high helical content and was twice more potent than the hit peptide 3ac in the cellular RSV-enhanced GFP (RSV-eGFP) assay (data not shown).

**TABLE 2:**
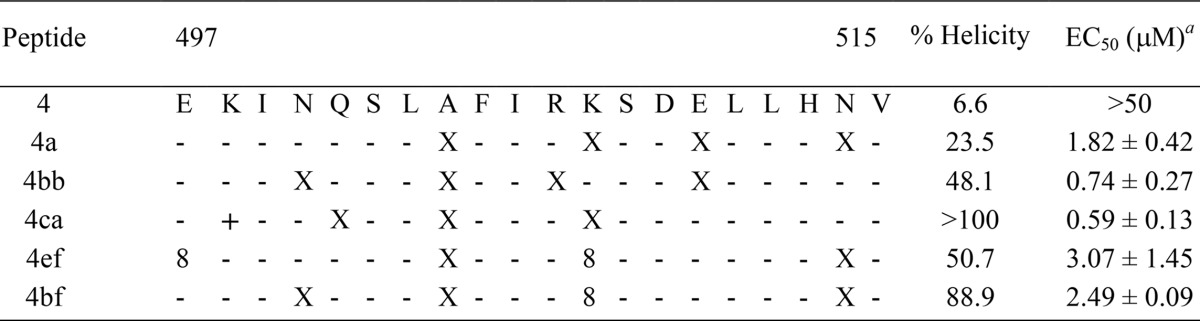
Design, alpha-helicity, and inhibitory activity of double-stapled peptides in Hep-2 cells

^a^The EC_50_ inhibitory activity was assessed using an RSV-Cherry virus at an MOI of 0.2. The peptide and the virus were removed following the infection, and fresh peptide was immediately added to maintain the pressure of inhibition. Each experiment was performed at least twice in duplicate.

Encouraged by these results, we engineered other double-stapled peptide analogs of peptide 4 by combining a staple of subdomain 2 with a staple of subdomain 3 (Table 2). The choice of the staples was based on the α-helical content observed for the single-stapled peptides. For example, peptide 4ef combines the staple of peptide 2e and the staple of peptide 3f. The CD spectra of peptide 4bb, 4bf, and 4ef appeared to display similar negative values at 208 nm and 222 nm, with α-helical contents in a range similar to that of peptide 3ac (Table 2 and Fig. 4A). The CD spectra of peptides 4ca and 4bf showed an unusually high helical content for peptides of this size in an aqueous environment, suggesting that they are fully folded into α-helices. To assess the oligomeric state of peptide 4ca, temperature-dependent CD spectra were measured at peptide concentrations of 25 and 50 μM. The temperature was increased from 25°C to 90°C in 5°C increments. No significant conformational change was observed during the course of this temperature ramping (T ramp) experiment for both peptide concentrations (see the supplemental material); furthermore, the CD curve of the sample cooled at room temperature after being heated at 90°C was very similar to the CD curve of the unheated sample. These data suggest that the α-helical structure of peptide 4ca is stable under denaturing conditions and that the peptide does not aggregate. Next, HEp-2 cells were infected with a recombinant RSV expressing the mCherry reported gene (24), and the inhibitory activity of the double-stapled peptides was quantified by fluorescence readout. We found that peptides 4bb and 4ca were more potent inhibitors (50% effective concentrations [EC_50_], 0.74 μM and 0.59 μM, respectively) than peptide 4a (Fig. 4B), whereas peptides 4ef and 4bf were somehow less active (EC_50_, 3.07 μM and 2.49 μM, respectively [Table 2]). In comparison, T118 displayed an EC_50_ of 6.33 μM under our conditions (data not shown). These data show that the insertion of two staples in peptides 4ca and 4bb resulted in increases of antiviral potency of 11- and 12-fold, respectively, despite the reduction of size from 35 aa in T118 to 20 aa in the peptide 4 stapled analogs.

### Identification of residues critical for the interaction of double-stapled peptides 4 with trimeric HR1.

**FIG 5:**
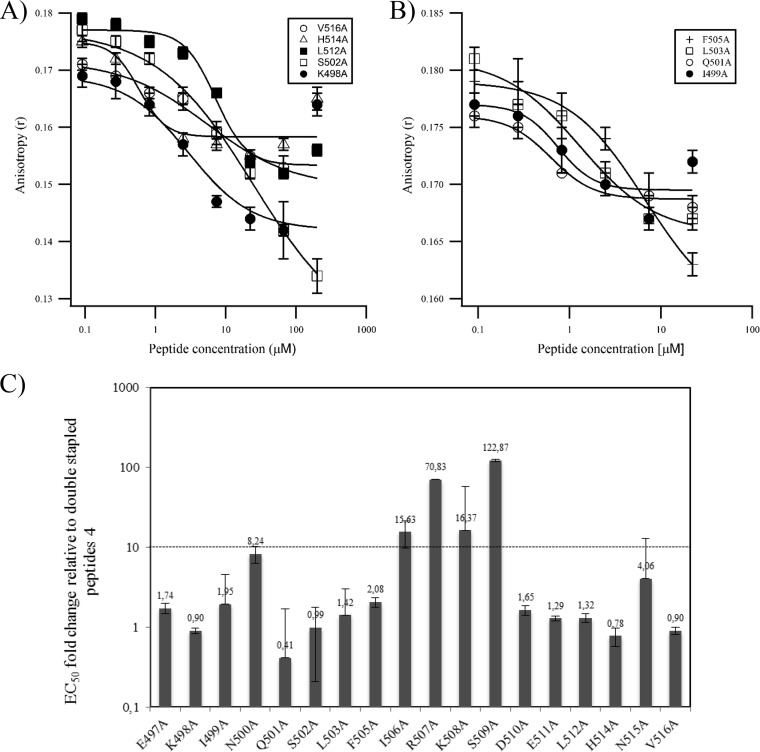
Ala-scanning mutagenesis of peptide 4. (A and B) Competitive fluorescence polarization assay of Ala mutants displaying a delta FP value in the range of 0.3 to 0.6 (error bars are standard deviations from duplicates) (A) and Ala mutants displaying a delta FP value in the range of 0.2 (B). (C) EC_50_ fold changes of peptide mutants relative to wild-type stapled peptides. The number above each bar chart is the EC_50_ fold change value. This experiment was repeated twice in duplicate; the first time used 2-fold serial dilutions of the variant peptides (11 dilutions) to enable EC_50_ determination, and the second time used a fixed peptide concentration of 5 μM to confirm the overall trend observed in the first experiment. Error bars are the standard deviations obtained upon fitting the first-experiment data with Igor Pro as described in Materials and Methods.

To investigate the contribution of each amino acid of peptide 4 to antiviral potency, we performed an alanine scanning mutagenesis experiment. Each amino acid of peptide 4bb was replaced by an alanine, except at positions 500, 507, and 511, which cannot be replaced since they are used for the stapling of peptide 4bb; for these residues, Asn^500^, Lys^507^, and Glu^511^ were replaced by Ala in peptide 4a and in peptide 4ca, respectively (Table 3). The effect of Ala substitution was first analyzed by UV circular dichroism spectropolarimetry. No major conformational change was observed, all the variants retaining a circular dichroism signature characteristic of a α-helix. The α-helical content of these variants is overall similar to that of peptide 4a, 4bb, or 4ca, except the I499A, Q501A, S502A, and L503A variants, which appeared to contain somehow less α-helical content (Table 3). The inhibitory activity of the Ala substitution peptides was also tested in the 5HB biochemical competition assay. As shown in Fig. 5A and B, 9 of the 17 variants were capable of blocking the increase of FP to an extent similar to that of 4bb. No signal could be reliably measured with the 8 remaining variants (data not shown). Last, each variant was tested in the viral cellular infectivity assay using an 8-fold serial dilution, and the EC_50_ inhibitory values of the variants were plotted relative to the EC_50_ of the nonsubstituted stapled peptide 4bb or 4a (Fig. 5C). In our analysis, an EC_50_ change of >10-fold is considered to reflect a substitution having a significant impact on antiviral activity. We found that only the I506A, R507A, K508A, and S509A substitutions led to a significant decrease of the inhibitory activity of the stapled peptide in cell infection assays. These data are consistent with the results of the 5HB biochemical competition assay, since none of these variants were able to compete with the binding of the fluorescently labeled T108 peptide to recombinant 5HB.

**TABLE 3:**
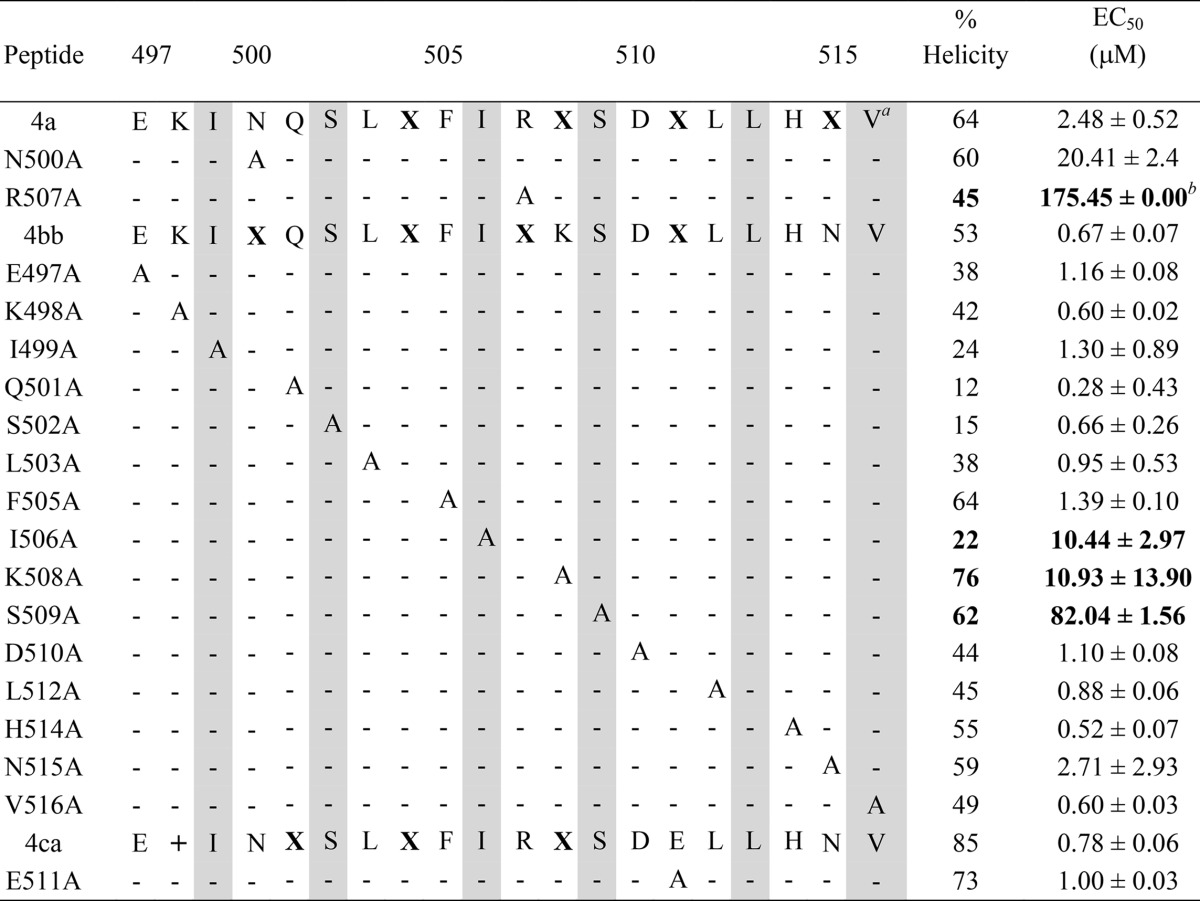
Ala-scanning mutagenesis of double-stapled peptide 4 analogs

^a^The shaded area shows the HR2 amino acid that makes hydrophobic contact with trimeric HR1.

^b^The numbers in bold refer to the mutant peptides that lose >10-fold inhibitory activity relative to their double-stapled peptide 4 analogs.

**TABLE 4:** Protease resistance of double-stapled peptides

Peptide	Half-life (h)	
	Chymotrypsin	Trypsin
4	0.08	0.08
4a	74.7	1.3
4bb	50.1	0.3
4ca	179.9	38.0
4ef	ND^*a*^	ND
4bf	ND	ND
T118	ND	ND
RSV-SAH_BD_ (Z)	7.4	0.08
RSV-SAH_BD_ (E)	6.5	0.02

a ND, not determined.

Altogether, these results reveal that 4 residues within the sequence of peptide 4 are critical for antiviral activity and should therefore be conserved in a medicinal chemistry effort to improve the inhibitory activity of the peptides. It is noteworthy that, among the 9 variants that were active in the FP assay, none was able to increase the potency of the peptides. The EC_50_ increase observed for the Q501A variant is statistically not significant.

### Comparison of the inhibitory activity of double-stapled peptides 4bb and 4ca with SAH-RSVF_BD_.

**FIG 6:**
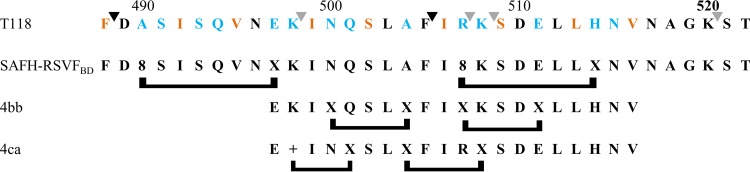
Schematic representation of the lead double-stapled peptides 4bb and 4ac and comparison with SAH-RSV_BD_. The T118 sequence identified by Lambert et al. (12) is depicted with the residues required for hydrophobic interactions with the trimeric HR1 coiled coils (8) in orange and the residues located at the interface of the hydrophobic and the hydrophilic face of HR2 in blue. The brackets below the peptides indicate the positions of the staples. +, R-pentenyl alanine; X, S-pentenyl alanine; 8, R-octenyl-alanine. The black and gray arrows above the T118 sequence refer to the chymotrypsin and trypsin cleavage sites.

**FIG 7:**
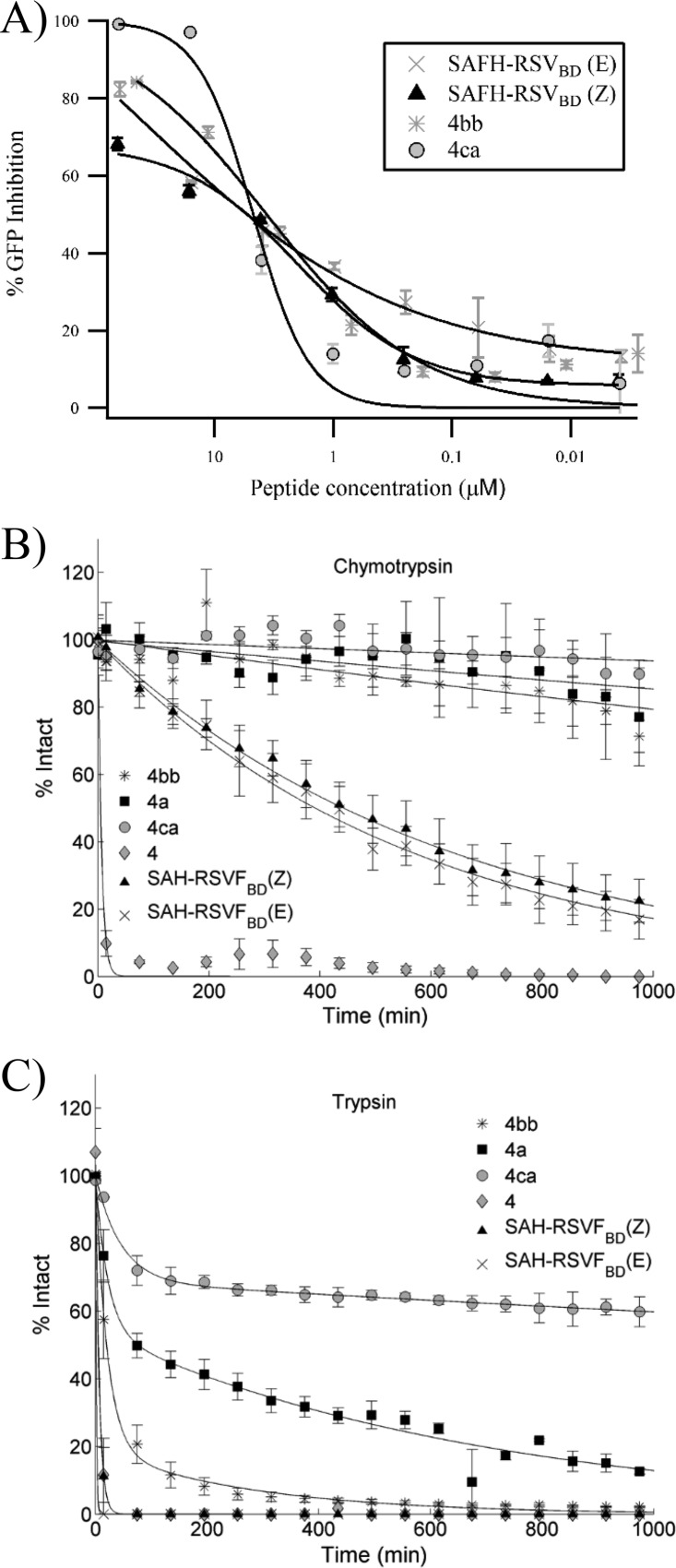
Comparison between the double-stapled peptides selected in this study and SAH-RSV_BD_ (18). (A) Effects of peptides on RSV-GFP infection of A549 cells. (B and C) Chymotrypsin and trypsin resistance profiles of the double-stapled peptides 4a, 4bb, and 4ca compared to the unstapled analog peptide 4, and the SAH-RSVF_BD_ Z and E isomers. The percent intact values were calculated as described in Materials and Methods. The mean percent intact values and standard errors of the means (SEM) were calculated and plotted from 3 experiments. These data were fitted as an exponential decay curve in MatLab and used to calculate each peptide degradation half-life.

In the course of our study, Bird et al. reported novel double-stapled peptides derived from the T118 peptide sequence capable of inhibiting RSV fusion (18) (Fig. 6). The best candidate in that work, SAH-RSVF_BD_, has been thoroughly characterized, including *in vivo* proof of concept: this peptide has been shown to prevent nasal and pulmonary RSV infection in BALB/c mice. Because SAH-RSVF_BD_ is significantly longer than our peptide 4 series (35-mer versus 20-mer) (Fig. 6), we decided to compare our best candidates, 4bb and 4ca, with SAH-RSVF_BD_. Peptide SAH-RSVF_BD_ was synthesized by solid-phase peptide synthesis (SPPS) in our laboratories. Unexpectedly, we observed two isomers of identical mass during the analysis of the crude material, which most likely result from the formation of two isomers at the staple olefinic bond (25). This isomerization has not been reported by the authors. We purified both isomers and arbitrarily assigned these isomers as SAH-RSVF_BD_ (Z) and RSVF_BD_ (E). The identity of the two isomers was confirmed by ultraperformance liquid chromatography (UPLC), electrospray mass spectrometry (ES-MS), and amino acid analysis. However, when the inhibitory activities of SAH-RSVF_BD_ (Z) and (E) isomers were tested in our cellular viral infection assay, we found that both isomers displayed a potency similar to that of peptides 4bb and 4ca (Fig. 7A).

Next, we compared the propensity of peptides SAH-RSVF_BD_, 4bb, and 4ca to resist to protease degradation in a proteolytic stability assay that was developed previously (17). This is an important parameter to assess for the development of peptide therapeutics. Peptides 4bb, 4ca, and 4a, and the unstapled analog peptide 4, as well as both SAH-RSVF_BD_ isomers, were treated with chymotrypsin or trypsin, and the samples were analyzed by LC/MS to quantify the reaction products over time. As expected, all stapled peptides were significantly more resistant to proteolytic degradation than the native peptide 4, which is fully degraded within 10 min (Table 4). As can be seen in Fig. 7B and C, our short stapled-peptide leads were highly resistant to proteolysis. In particular, peptide 4ca displayed half-lives of 180 h and 38.5 h against chymotrypsin and trypsin, respectively. In comparison, both SAH-RSVF_BD_ isomers were more susceptible to proteolytic degradation, with half-lives of approximately 7 h and 5 min against chymotrypsin and trypsin, respectively. Altogether, these results suggest that our lead peptide 4ca could be more stable overtime *in vivo* than SAH-RSVF_BD_.

### Intranasal administration of peptide 4ca inhibits RSV infection in BALB/c mice.

**FIG 8:**
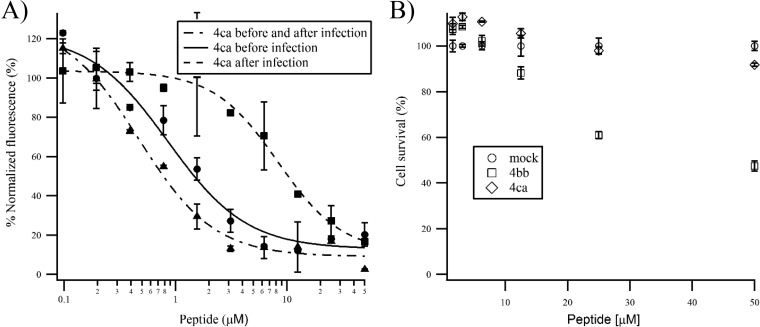
Optimization of peptide 4ca administration in mice. (A) *In cellula* inhibition assays. rHRSV-Cherry was preincubated for 2 h with or without peptide 4ca, and the mixture was then added to HEp-2 cells. After infection, the supernatant was changed against regular medium or medium supplemented with peptide 4ca at the same concentrations as those used during the infection. Viral replication was quantified by measuring the mCherry fluorescence at 48 h p.i. and normalized based on the fluorescence of infected and untreated cells. (B) Cytotoxicity assay. HEp-2 cells were incubated in the presence of serial dilutions of peptides, and cell survival was quantified using the CellTiter-Glo Luminescent cell viability assay 24 h after the addition of the peptides. The signal was normalized based on the luminescence obtained for untreated cells. Error bars are standard deviations from duplicates.

To seek the best conditions to administer the stapled peptide to mice, the inhibition of RSV infection by peptide 4ca was first tested in HEp-2 cells under three different experimental conditions: (i) addition of 4ca following the infection of cells with the recombinant human RSV (rHRSV)-mCherry (multiplicity of infection [MOI], 0.2); (ii) preincubation of the virus with peptide 4ca prior to cell infection; (iii) preincubation of the virus with peptide 4ca prior to cell infection and addition of a fresh solution containing peptide 4ca in the cell culture medium after infection. To measure the inhibitory activity of the peptide, the mCherry red fluorescence was quantified 48 h postinfection (p.i.). Our results clearly show that peptide 4ca inhibits RSV replication under the three conditions tested, although preincubation of the virus with the peptide was more efficient than the treatment after RSV infection, with EC_50_s of 0.86 μM and 8.3 μM, respectively (Fig. 8A). Additionally, we found that maintaining the presence of the peptide in the medium after infection improved its antiviral efficacy (EC_50_ = 0.47 μM). It is noteworthy that no cellular toxicity was detected (Fig. 8B).

**FIG 9:**
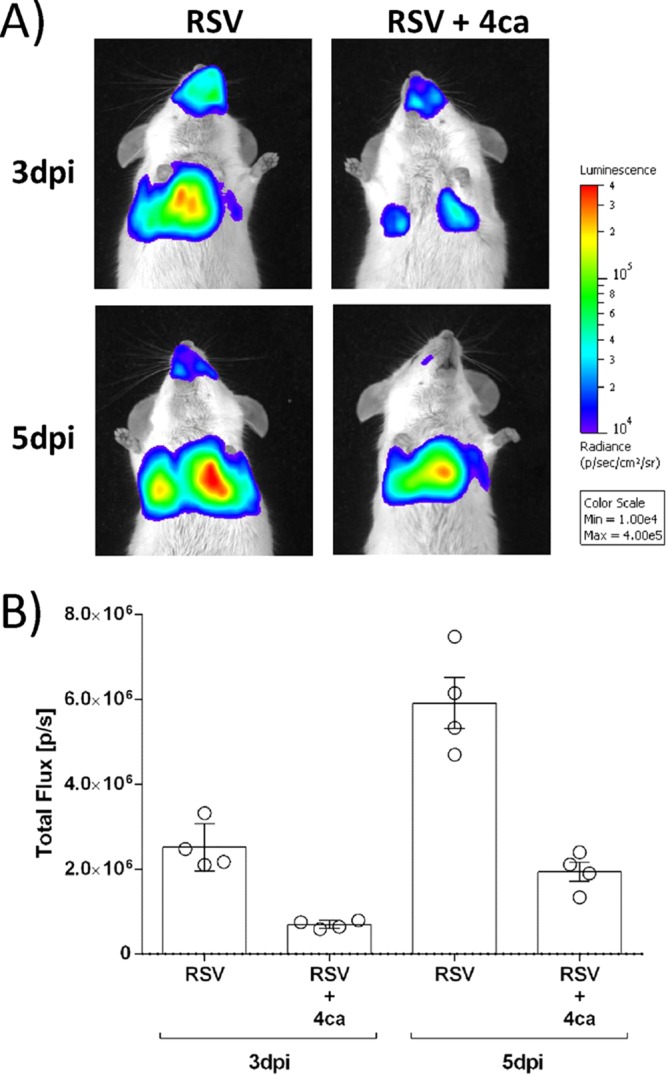
*In vivo* inhibition of RSV infection by intranasal treatment of mice with peptide 4ca. (A) Groups of 4 BALB/c mice were treated at day 0 with either 50 μl of PBS or peptide 4ca in PBS (50 μl at 50 μM) and infected 10 min later by rHRSV-Luc (5 × 10^4^ PFU per mouse). Bioluminescence was then measured at days 3 and 5 p.i. by intranasal injection of 50 μl of d-luciferin (200 mM). Capture of photon emission from the chest was performed using the IVIS system. Ventral views of one mouse representative of the results are shown. The scale on the right indicates the average radiance: the sum of the photons per second from each pixel inside the region of interest/number of pixels (p s^−1^ cm^−2^ sr^−1^). (B) Comparison of bioluminescence of infected mice untreated or treated with peptide 4ca. Luciferase activities were quantified for each mouse using Living Image software. Luciferase activity is expressed as photons per second (p s^−1^). Each data point represents the mean ± SEM (*n* = 4). The statistical significance of differences was calculated using Student's *t* test.

**FIG 10:**
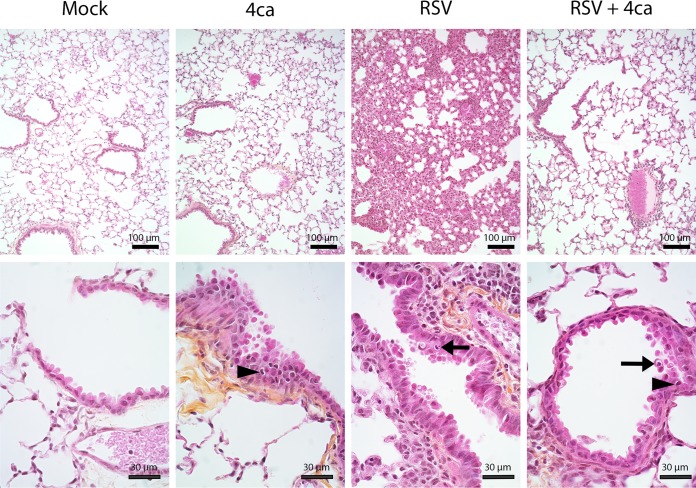
Histopathological analysis of lungs of mice treated with peptide 4ca. Groups of 4 BALB/c mice were treated at day 0 either with 50 μl of PBS or with peptide 4ca in PBS (50 μl at 50 μM) and infected or not 10 min later by rHRSV-Luc (5 × 10^4^ PFU per mouse), as described for Fig. 8. Mice were sacrificed 7 days p.i., and sections of fixed lungs were subjected to HES staining. Upper and lower panels show representative lung sections at low and high magnifications, respectively. Compared to mock-inoculated animals, no changes were noticed in the lung parenchyma of 4ca-inoculated mice, except for the presence of a few inflammatory cells (arrowhead) in the bronchial epithelium (transepithelial exocytosis). Whereas a diffuse interstitial pneumonia is observed in RSV-inoculated mice, no lung parenchyma lesion is present in tissue of mice treated with peptide 4ca and RSV. Several degenerating cells, exhibiting a pyknotic nucleus inside the bronchial epithelium, were observed (arrow) for all the infected animals.

Next, we assessed the antiviral activity of peptide 4ca in mice using the conditions optimized above. In order to track the infection, we used the recombinant virus rHRSV-Luc, which was previously shown to allow *in vivo* imaging studies of RSV replication and spreading and constitutes a powerful and convenient tool for the evaluation of drug efficiency against RSV in the mouse model (24). BALB/c mice of 8 weeks of age (*n* = 4) were treated by intranasal administration of peptide 4ca (50 μl at 50 μM in phosphate-buffered saline [PBS]) or PBS alone, followed by intranasal (i.n.) inoculation with a single dose of rHRSV-Luc (5 × 10^4^ PFU). Mice were anesthetized at day 3 p.i. and observed alive using the IVIS imaging system 5 min after i.n. injection of d-luciferin. As shown in Fig. 9A, the bioluminescence was clearly reduced both in the snout and in the lungs of the treated mice compared to control mice. At day 3 p.i., a second dose of the peptide was administered to the mice, and the bioluminescence was measured at day 5 p.i. A 3-fold decrease of viral infection was observed in mice treated with 4ca compared to untreated mice (Fig. 9B). At day 7 p.i., a time corresponding to the end of the infection in mice, the animals were sacrificed and the lungs were collected in order to perform a histological analysis. The aim of this histological analysis was to validate indirectly the efficiency of peptide 4ca by the observation of the clinical hallmarks of RSV infection. Additionally, we wished to detect any potential toxicity of peptide 4ca due to the treatment at the tissue scale and the potential immune response induced upon treatment. As shown in Fig. 10, no histological changes were noticed in the lung parenchyma between mock- and 4ca-inoculated animals, suggesting the absence of toxicity of 4ca in the lungs. Infection by RSV was responsible for a multifocal extensive marked interstitial pneumonia characterized by a diffuse thickening of the alveolar walls, by mononuclear cell infiltration, and by a bronchus-associated lymphoid tissue (BALT) hyperplasia. In contrast, no interstitial pneumonia was observed after infection by RSV upon inoculation of 4ca, although some BALT hyperplasia and the presence of some degenerating cells inside the bronchial epithelium were observed. These observations confirm the antiviral effect of peptide 4ca.

On the other hand, we detected the presence of few inflammatory cells in the bronchial epithelium upon treatment with 4ca, thereby suggesting that peptide 4ca may induce a weak transepithelial exocytosis and thus a possible induction of the immune system. To investigate this further, peptide 4ca was administered to noninfected animals according to the same protocol. The blood of mice was collected 1 month after the first inoculation, and the presence of anti-4ca antibodies in the sera of these animals was analyzed by enzyme-linked immunosorbent assay (ELISA). Peptide 4ca was immobilized on 96-well plates, followed by incubation with the mice sera, and antibodies raised against peptide 4ca were sought with anti-IgG1, anti-IgG2a, and anti-IgA. As a positive control, we used our recombinant His-tagged 5HB protein and an anti-His antibody to verify that peptide 4ca was successfully immobilized onto the plate. The presence of peptide 4ca was confirmed through its interaction with 5HB, but no antibodies specific to 4ca could be detected in the sera (data not shown). These results suggest that the recruitment of immune cells in the lungs of treated mice was not due to the induction of an immune response directed against 4ca.

## DISCUSSION

Here we have performed a stapled-peptide scan of 16-mer overlapping sequences derived from the HR2 domain of the RSV F protein. We identified novel double-stapled peptides, which presumably interfere with the formation of the six-helix bundle postfusion structure required for viral cell entry. Using Ala-scanning mutagenesis experiments, we have identified the residues required for inhibition. We have shown that peptide 4ca can be administered to BALB/c mice with no signs of toxicity or immunogenicity and prevents nasal and pulmonary RSV infection.

**FIG 11:**
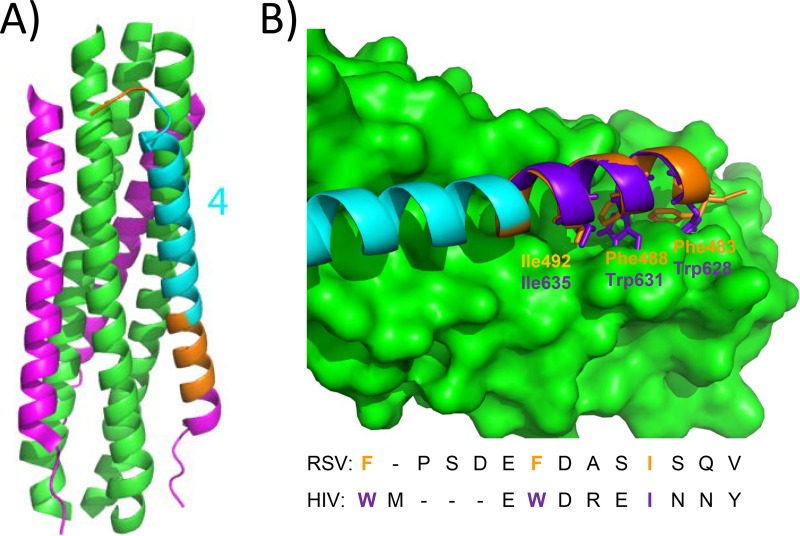
(A) Schematic representation of the F six-helix bundle in the postfusion conformation. The HR1 trimeric coiled coils are in green; two of the 3 HR2 helices interacting with HR1 are in purple; in the third HR2 helix, peptide 4 is highlighted in cyan, and SAH-RSV_BD_ spans the region highlighted in gold from the N terminus to the C terminus of peptide 4. The amino termini of the HR2 helices point toward the bottom of the page and those of the HR1 helices point toward the top. (B) Superimposition of the RSV and HIV HR2 sequences bound to trimeric HR1 as observed in the X-ray postfusion structure (PDB codes 1G2C and 1AIK, respectively). The HR2 N-terminal sequence interacting with the deep hydrophobic groove is colored in gold for RSV and in purple for HIV. The HIV and RSV sequences are aligned at the bottom of the figure, and the amino acids required for the binding are also colored in gold and purple.

Of all the single-stapled peptides that were screened, none was found to inhibit viral fusion. These data suggest that the binding energy of the six-helix bundle postfusion complex is too high to be disrupted by short single-stapled peptides, unlike what has been demonstrated for other protein targets involved in the Notch (26) or the B-cell lymphoma 2 (BCL-2) signaling pathways (27). The double-stapled peptides identified here are derived from peptide 4 (colored in cyan in Fig. 11A) and are located at the C terminus (aa 497 to 516) of the HR2 α-helical domain (aa 485 to 515) (8). The N-terminal HR2 α-helical segment lacking in peptide 4 (colored in orange, with one turn in magenta in Fig. 11A) contains the key determinants Phe 483, Phe 488, Ile 492, and Val 495, which are required for binding to a deep hydrophobic groove located at the C terminus of the HR1 trimeric coiled coil. Superimposition of the RSV and the HIV postfusion crystal structures of the N- and C-terminal repeat domains has shown that this binding pocket is almost identical to the HIV gp41 binding pocket (Fig. 11B), also commonly referred as the HIV's deep pocket (8, 28). This pocket has drawn considerable attention in the field of HIV antiviral drug discovery, as it has been postulated to be a good site of intervention for small molecules. Additionally, this pocket has also been used recently to develop the next generation of HIV fusion inhibitors, because the peptide drug enfurvitide also lacks this HR2 N-terminal segment, similarly to peptide 4 (29). In the field of RSV, small molecules binding to this site have been identified (30, 31), and peptidomimetics have been rationally designed to target this hydrophobic pocket (15, 32). In contrast to these precedents, our efforts to inhibit viral fusion through targeting this pocket with stapled peptides (subdomain 1 in Fig. 1B) were not successful. The single-stapled peptides 1d and 1f contain an all-hydrocarbon staple at the same positions as those that were used to insert a bis-lactam bridge in mimetic 1, a compound reported to display an EC_50_ of 36 nM in an RSV antiviral assay (15). We attempted to synthesize the double-stapled peptide corresponding to mimetic 1, but the synthesis failed because we were not able to couple two adjacent pentenyl-alanine residues, presumably due to hydrophobic- and sterical-hindrance reasons. The authors further reported that the replacement of Phe483 and Pro484 with 1-naphtylalanine can improve peptide inhibition 1,000-fold (32). Here again, we made the same substitution in peptide 1e, but no improvement of peptide inhibition was observed (data not shown).

The Ala scan of stapled peptides 4 led to the findings that the Ile506, Arg 507, Lys508, and Ser509 substitutions are not tolerated. Residues 506 to 509 are located in a region reported to be a patch of polar contacts between HR2 and HR1 (8). The loss of binding of the Ile506Ala and Ser509Ala variants is not surprising since Ile506 is involved in a key hydrophobic contact with HR1 and Ser-509 Oγ forms an elaborate hydrogen bond network with Thr-174 Oγ1, Ala-170 O, and Asn-175 Nδ2 from the HR1 helices. However, the loss of binding of the Arg507Ala and Lys508Ala variants is unexpected, as these residues are located at the hydrophilic noninteracting face of the HR2 peptide. We could not find any rational explanation for this effect upon inspection of the crystal structure, except the fact that Arg507 is involved in an ionic interaction with Glu511. However, this interaction is unlikely to be the cause for the loss of binding, since Glu511 is used for the stapling in the Arg507Ala variant and the Glu511A variant retains its inhibitory activity (Table 3). It cannot be excluded that Arg507 and Lys508 are involved in other ionic interactions that are not seen in the crystal structure. Therefore, other types of salt bridges may be investigated at these sites to improve antiviral potency, similarly to the salt bridge strategy that has been used in HIV to improve the stability of peptidic fusion inhibitors (33).

The peptides identified here are significantly shorter than the double-stapled peptide SAH-RSV_BD_ reported by Bird et al. (18). SAH-RSV_BD_ spans three additional helical turns at the N terminus of peptide 4 and an unfolded area at the C terminus of the peptide (Fig. 11A, in orange). The alpha-helical structure of SAH-RSV_BD_ is stabilized with two “i, i + 7” staples, while the peptides 4bb and 4ac are constrained with two “i, i + 4” staples and with “i, i + 3” and “i, i + 4” staples, respectively. Remarkably, the difference in length does not result in a decrease of inhibition of viral fusion, as would be expected given that these peptides interact with a smaller surface area on the trimeric HR1 target protein. Furthermore, our peptides are less susceptible to proteolysis and are therefore expected to be more stable *in vivo*. Most likely, the closer proximity of the staples in peptides 4bb and 4ca resulting in enhanced alpha-helical packing provides less accessibility to the chymotrypsin and trypsin serine proteases.

Our data show that the minimal domain peptide 4 is a valuable means to prevent the formation of the postfusion 6HB complex required for RSV F fusion. From our mutagenesis study, we conclude that many residues of peptide 4 contributing to the interface interaction with HR1 can tolerate chemical modification and can thereby form the basis for a medicinal effort toward improving the antiviral properties of this peptide series. The successful intranasal delivery of peptide 4ca to mice and inhibition of RSV infection suggest that an inhalable therapy may be developed to treat RSV-infected individuals with such molecules. However, the development of an efficacious inhalable treatment is expected to be difficult in young infants, because of the challenge in treating the pediatric population with this type of formulation (34). The ongoing clinical trial of ALX171 in infants may pave the way for such a strategy. Alternatively, an injectable long-acting formulation may be envisioned.

## MATERIALS AND METHODS

### Materials.

Fmoc (9-fluorenylmethoxy carbonyl) amino acids and coupling reagents were purchased from Aapptec, Novabiochem, and Bachem. The nonnatural olefin-containing amino acids were purchased at Okeanos Tech. Co., Ltd. Solvents were purchased from Acros, Biosolve, and Sigma-Aldrich.

### Cell culture.

A549 (ATCC CCL-185) cells were maintained in Dulbecco's modified Eagle medium (DMEM) supplemented with 10% fetal calf serum (FCS). HEp-2 (ATCC CCL-23) cells were maintained in Eagle's miminum essential medium (EMEM) supplemented with 10% FCS, 2 mM l-glutamine, and penicillin-streptomycin solution. The cells were grown in an incubator at 37°C in 5% CO_2_. Cytotoxicity assays were done with the CellTiter-Glo Luminescent cell viability assay (Promega).

### Peptide synthesis.

Peptides were synthesized by solid-phase peptide chemistry on a Rink Amide AM resin LL (100 to 200 mesh; Novabiochem) using an Apex 396 Automated Multiple Peptide Synthesizer (Aapptec) at a 50-μmol scale. Each coupling was performed with HCTU [*O*-(6-chlorobenzotriazol-1-yl)-*N*,*N*,*N*′,*N*′-tetramethyluronium hexafluorophosphate] and diisopropyldiethylamine (DIEA) in *N*-methyl-2-pyrrolidone (NMP) for the standard amino acids. For the coupling following the nonnatural olefinic amino acids, HCTU was replaced by PyClock or HATU (1-[bis(dimethylamino)methylene]-1*H*-1,2,3-triazolo[4,5-*b*]pyridinium 3-oxid hexafluorophosphate). Following final Fmoc deprotection and N-terminal acetylation, the metathesis was performed under constant nitrogen degassing, in a 2-ml solution containing 10 mM 1st generation Grubbs' catalyst in dichloroethane (DCE). The metathesis was performed twice for 2 h at room temperature. Peptides were deprotected and cleaved from the resin with a cleavage cocktail consisting of trifluoroacetic acid (TFA)-triisopropylsilane (TIS)-H_2_O (95:2.5:2.5) for 1.5 h. Crude peptides were analyzed by UPLC/MS (Waters Acquity Ultra Performance LC/Micromass Quattro micro API) on an Acquity UPLC BEH C_18_ column (1.7 μl, 1.0 by 50 mm) and purified by high-performance liquid chromatography (HPLC) preparative (Waters 2777 sample manager, Waters 2545 binary gradient module, Waters 2487 Dual λ Absorbance Detector) using a Waters C18 Xbridge PreShield RP18 column (19 by 100 mm; diameter particle size, 5 μm). Samples were lyophilized and quantified with the Qubit 2.0 fluorometer (Life Technologies).

### CD spectroscopy.

The circular dichroism spectra were acquired on a Chirascan spectropolarimeter. The samples were prepared in 10 mM phosphate buffer, pH 7.5, at a peptide concentration of 50 μM. Data were recorded at 25°C by step scan from 180 nm to 260 nm in a 0.5-mm path length quartz cell using 1-nm wavelength increments and a response time of 1 s. The data were converted to per residue molar ellipticity units [θ] (degree per square centimeter per decimole per residue) and smoothed using the Igor Pro software. The percentage of helicity was calculated as described previously (35).

### Cloning, expression, and purification of 5HB.

The coding sequence of 5HB was designed as described previously and *de novo* synthesized by Genscript (21). The HR1 (aa 126 to 182) and HR2 (aa 476 to 524) coding sequences from wild-type RSV A2 strain F (36) were codon optimized for overexpression in Escherichia coli and cloned at NdeI/BamHI restriction sites of pET-15b to generate the expression plasmid for 5HB. The 5HB expression construct was transformed into Escherichia coli BL21(DE3). Cells were grown at 37°C to an optical density at 600 nm of 0.8 followed by induction with isopropyl-1-thio-β-d-galactopyranoside at a final concentration of 0.5 mM for 12 to 15 h. Cells were harvested by centrifugation, washed twice with PBS, and resuspended in 20 ml of PBS (pH 7.4), 500 mM NaCl, and 1% Triton X-100 supplemented with complete protease inhibitor. Cell suspensions were disrupted by sonication. Cell debris was removed through ultracentrifugation at 18,000 × *g* for 1 h at 4°C, and clarified cell lysate was mixed with 1 ml of nickel-nitrilotriacetic acid (Ni-NTA) agarose beads (Qiagen) preequilibrated with 20 ml of lysis buffer. The suspension was agitated for 1 h at 4°C and loaded onto a 5-ml polypropylene column (Qiagen). The column was washed twice with 8 ml of PBS (pH 7.4), 500 mM NaCl, 1% Triton X-100, and 100 mM imidazole. Proteins were eluted with 5 × 500 μl of a buffer containing PBS (pH 7.4), 300 mM imidazole, and 500 mM NaCl. The imidazole was removed by dialysis, and protein purity was assessed by SDS-polyacrylamide gel electrophoresis followed by Coomassie blue staining. Protein concentration was determined with the Qubit 2.0 Fluorometer (Life Technologies).

### 5HB fluorescence polarization assay.

The T108 (12) peptide probe was synthesized by standard SPPS procedures using HCTU as a coupling reagent as described above. A 10-μl volume of recombinant 5HB protein in FP buffer (20 mM PBS [pH 7.4], 500 mM NaCl, 0.01% [vol/vol] Tween 20, and 0.05 mg/ml bovine gamma globulin) was preincubated for 10 min at room temperature with 10 μl of the appropriate concentration of the inhibitor, after which 10 μl of fluorescein isothiocyanate (FITC)-T106 was added and further incubated for 30 min at room temperature. The final concentrations of protein and probe were 50 nM and 2 nM, respectively. The fluorescence polarization assay was performed in 384-well plates using a Spectramax Paradigm (Molecular Devices), using excitation and emission wavelengths of 485 nm and 535 nm, respectively. The acquisition time was 700 ms, and the read height was 1 mm. All experiments were performed in duplicate. Resulting inhibition curves were fitted with a 4-parameter logistic equation using a nonlinear curve-fitting Levenberg-Marquardt algorithm implemented in Igor Pro (Wavemetrics, Tigard, OR, USA), *r* = *r*_0_ + {Δ*r*_max_/[1 + (IC_50_/*c*_inhibitor_)^*n*^]}, where *r* is the anisotropy, IC_50_ is the 50% inhibitory concentration, *c*_inhibitor_ is the inhibitor concentration, *r*_0_ is the anisotropy in the absence of recombinant 5HB protein, *n* is the Hill coefficient, and Δ*r*_max_ is the anisotropy change in the presence of a large excess of protein. In order to ensure robust fitting for some fits, *r*_0_ was kept constant at the value measured for the free tracer in buffer.

### HRSV-eGFP inhibition assay.

Serial 4-fold dilutions of inhibitors were mixed with eGFP-encoding RSV virus (23) with a volume of virus required to achieve approximately 50% of infection in medium containing 2% fetal calf serum. The resulting mixture was added to 24-well plates containing A549 cells. Cells were incubated for 2 h at 37°C, the medium was replaced, and cells were incubated for an additional 24 h. Cells were treated with 300 μl of trypsin for 10 min at 37°C, and the detached cells were diluted in 1 ml of culture medium. Following centrifugation at 1,700 rpm, the pellet was resuspended in 300 μl of PBS–1% fetal bovine serum (FBS) (vol/vol) and GFP-positive cells were counted by fluorescence-activated cell sorter (FACS) analysis. The GFP data were normalized to the fluorescence value observed with virus only. The resulting inhibition curves were fitted in Igor Pro as described above.

### rHRSV-mCherry inhibition assay.

HEp-2 cells were seeded at 5 × 10^4^ cells per well in a 96-well plate the day before the infection. Peptides were 2-fold serially diluted in dimethyl sulfoxide (DMSO) (11 dilutions) and then further diluted in EMEM without phenol red and preincubated for 15 to 20 min with rHRSV-mCherry (24) at a final dilution with an MOI of 0.2 (24). The cells were incubated with the virus-peptide mixtures for a period of 1.5 to 2 h. The medium was then replaced by EMEM without phenol red containing 2% fetal bovine serum and the same concentration of peptide as the one that was used during the infection step. Plates were incubated 48 h at 37°C, and the mCherry fluorescence was measured using a spectrofluorometer (Tecan infinite M200PRO) with excitation and emission wavelengths of 580 and 620 nm, respectively. Relative fluorescence was normalized by the fluorescence of nontreated infected cells. Noninfected HEp-2 cells were used as standards for fluorescence background levels. Each experiment was performed in duplicate and repeated at least twice. The resulting inhibition curves were fitted in Igor Pro as described above.

### Proteolytic stability assay.

*In vitro* proteolytic degradation was measured via LC-MS using an Agilent Infinity 1280 UHPLC system coupled to an Agilent quadrupole time of flight (QTOF) 6530 instrument with an Agilent Jet Stream electrospray ionization (AJS ESI) source (all from Agilent Technologies, Waldbronn, Germany). Five microliters of digested sample was injected onto a C_18_ column (Zorbax Eclipse Plus RRHD, 1.8 μm, 2.1 by 100 mm; Agilent Technologies, Waldbronn, Germany) equilibrated with solvent A (5% acetonitrile, 0.2% formic acid) and eluted with a flow rate of 0.4 ml/min over a 5 to 95% gradient of solvent B (95% acetonitrile, 0.2% formic acid) in 5 min, followed by 1.5 min at 95% and 0.5 min posttime. The AJS ESI source was operated with a capillary voltage of 4,000 V and a nozzle voltage of 600 V with a drying gas temperature of 325°C and flow rate of 10 liters/min, nebulizing gas pressure of 20 lb/in^2^, and a sheath gas temperature of 300°C and flow rate of 11 liters/min. Mass spectra were acquired in the positive-ion mode from 100 to 3,200 *m/z* at a rate of 1 scan per second in extended dynamic range (2 GHz) mode. The fragmentor, skimmer, and octopole RF voltages were set to 200, 60, and 750 V, respectively. Data were acquired and analyzed with the Agilent MassHunter Workstation (version B.05).

Peptide digestion was performed as follows: 10 μl of peptide at 0.29 mM in 50:50 acetonitrile-water was dissolved in 990 μl 100 mM Tris-HCl (pH 8.0) with 10 mM CaCl_2_. Following measurement of the undigested (*t*_0_) sample, 10 μl of chymotrypsin at 50 μg/ml in 100 mM Tris-HCl (pH 8.0) with 10 mM CaCl_2_ was added, and the sample was vortexed and placed immediately in the autosampler, set to 25°C. The level of intact peptide was quantified by serial injection over time. Integration of the intact peptide compound chromatogram was performed using the Agilent Molecular Feature Extraction (MFE) algorithm. The value of the fit at *t* = 0 min was used to calculate the percentage of the intact peptide at each point as follows: Percent intact (*t*) = [Vol (*t*)/Vol_*t*0_] × 100, where Vol is the total volume calculated using Find Compounds by Molecular Feature and Vol_*t*0_ is the value of the fit at time *t* = 0. A plot of the intact peptide MFE peak area over time gave an exponential decay curve, from which the half-life was determined using MatLab (Matrix Science).

### RSV infection of mice and intranasal treatment.

Female BALB/c mice were purchased from the Centre d'Elevage R. Janvier (Le Genest Saint-Isle, France) and were used at 8 weeks of age. Mice (*n* = 4 per group) were anesthetized with of a mixture of ketamine and xylazine (1 and 0.2 mg per mouse, respectively) and treated i.n. with 50 μl of peptide 4ca at 50 μM in PBS or with PBS alone for control mice. Ten minutes later, mice were infected i.n. with 50 μl of rHRSV-Luc (5 × 10^4^ PFU) (24). Three days postinfection (p.i.), mice were anesthetized for the *in vivo* luminescence measurement and treated i.n. a second time with 50 μl of peptide 4ca at 50 μM in PBS. Luminescence measurement was then performed at day 5 p.i.

### Histological analysis.

Mice were sacrificed at day 7 p.i., the chest cavity was opened, and the lungs were perfused intratracheally with 4% paraformaldehyde (PFA) in PBS. The lungs were then removed and immersed in 4% PFA for 12 h before transfer to 70% ethanol. The lungs were embedded in paraffin, and 5-μm sections were cut, stained with hematoxylin-eosin-saffron (HES), and evaluated microscopically. Qualitative histological changes were described and when applicable were scored semiquantitatively using a three-point scale from 0 to 2 (0, none; 1, mild; 2, marked), focusing on histological characterization of lesions (interstitial pneumonia, respiratory epithelial cell apoptosis, and hyperplasia) and inflammation.

### *In vivo* luminescence measurements.

Mice were anesthetized, and luminescence was measured 5 min following i.n. injection of 50 μl of PBS containing 0.75 mg kg^−1^ of body weight d-luciferin (Sigma). Photon emission of mock-infected and rHRSV-Luc-infected mice was measured using the IVIS 200 imaging system (Xenogen Corp.). Living Image software (version 4.0; Caliper Life Sciences) was used to measure the luciferase activity. Bioluminescence images were acquired for 1 min with f/stop of 1 and binning of 8. Digital false-color photon emission images of mice were generated, and photons were counted within a constant region of interest corresponding to the surface of the chest encompassing the whole-airway area. Photon emission was measured as radiance in photons per second per square centimeter per steradian (p s^−1^ cm^−2^ sr^−1^).

### Ethics statement.

The *in vivo* work of this study was carried out in accordance with INRA guidelines in compliance with European animal welfare regulation. The protocols were approved by the Animal Care and Use Committee at “Centre de Recherche de Jouy-en-Josas” (COMETHEA) under relevant institutional authorization (“Ministère de l'éducation nationale, de l'enseignement supérieur et de la recherche”), authorization number 2015100910396112v1 (APAFIS#1487). All experimental procedures were performed in a biosafety level 2 facility.

## Supplementary Material

Supplemental material
