# Supplementary material for: A Short Double-Stapled Peptide Inhibits Respiratory Syncytial Virus Entry and Spreading
Source: Antimicrob Agents Chemother. 2017 Mar 24;61(4):e02241-16. doi: 10.1128/AAC.02241-16 (PMC5365662; doi:10.1128/AAC.02241-16)
Supplement: Supplemental material [file supp_61_4_e02241-16__index.html]

Supplemental material 

# A Short Double-Stapled Peptide Inhibits Respiratory Syncytial Virus Entry and Spreading

## Supplemental material

- Supplemental file 1 -

  Supplemental Figure S1

  PDF, 136K
