# Supplementary material for: A Short Double-Stapled Peptide Inhibits Respiratory Syncytial Virus Entry and Spreading
Source: Antimicrob Agents Chemother. 2017 Mar 24;61(4):e02241-16. doi: 10.1128/AAC.02241-16 (PMC5365662; doi:10.1128/AAC.02241-16)
Supplement: Supplemental material [file AAC.02241-16_zac004176043s1.pdf]

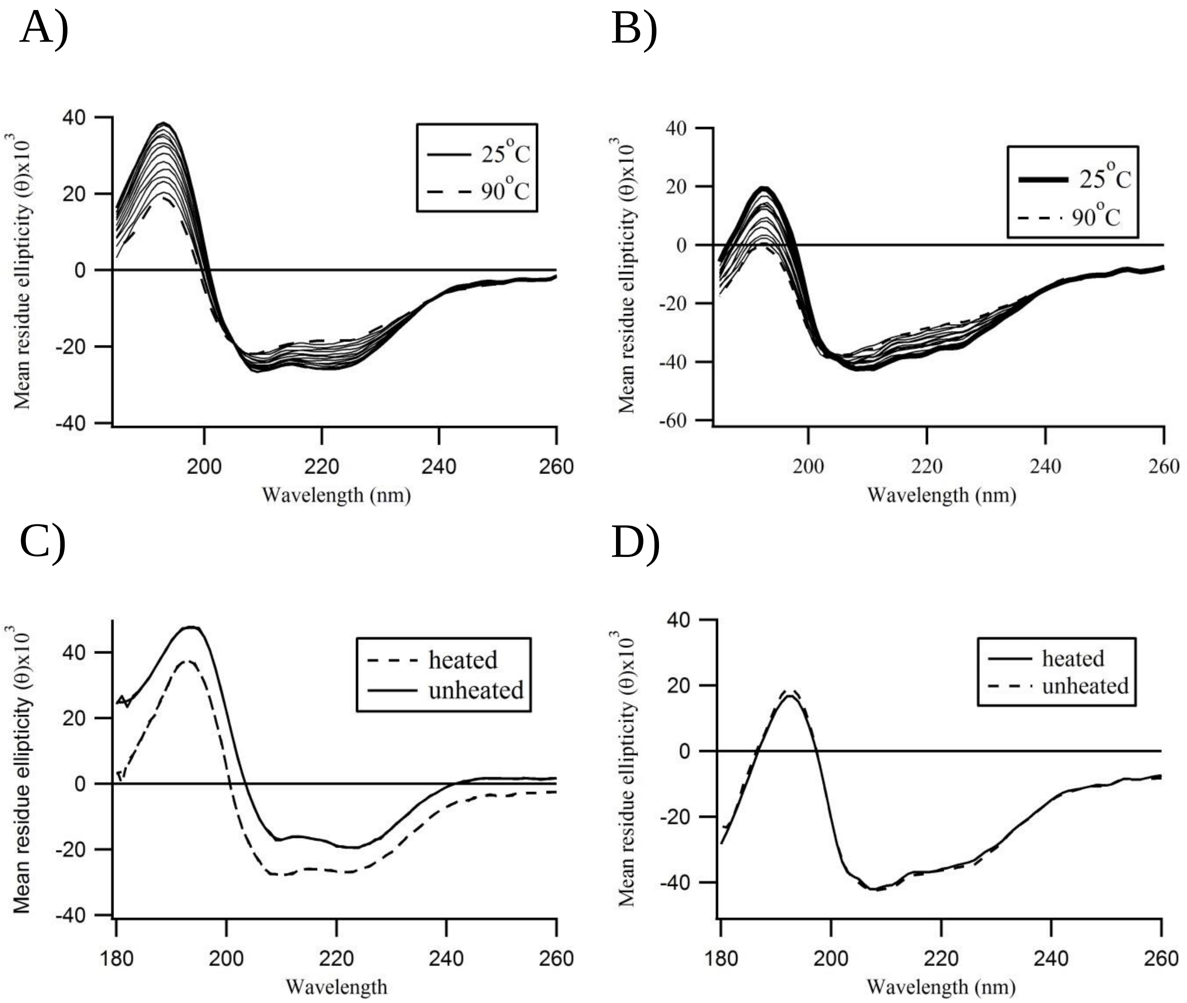

Figure S1. Temperature dependent circular dichroism spectra of peptide **4ca** at a peptide concentration of A) 50 μM and B) 25 μM. The cell holding the sample was heating at the desired temperature and the CD spectra was measured as described in material and methods. The temperature was increased from 25°C to 90°C by 5°C increments. Panel C) and D) are the CD curve performed at 25°C prior to increase the temperature (unheated) and after letting the sample heated at 90°C (heated) cool down to 25°C.
